# Development of a metasurface-based slot antenna for 5G MIMO applications with minimized cross-polarization and stable radiation patterns through mode manipulation

**DOI:** 10.1038/s41598-024-58794-1

**Published:** 2024-04-05

**Authors:** Hamed Hamlbar Gerami, Robab Kazemi, Aly E. Fathy

**Affiliations:** 1 Faculty of Electrical and Computer Engineering, University of Tabriz, Tabriz, 5166616471 Iran; 2 Department of Electrical Engineering and Computer Science, University of Tennessee, Knoxville, 37996 USA

**Keywords:** Characteristic mode analysis (CMA), Metasurface antenna, MIMO antenna, Suppressed cross-polarization (XP), 5G mm-wave, Engineering, Electrical and electronic engineering

## Abstract

This paper presents an approach for designing metasurface antennas using the characteristic mode analysis method for 5G mm-wave multiple input–multiple output (MIMO) systems. The proposed metasurface antenna consists of a 3 × 3 array of modified patches with additional slits and stubs, which are fed by a coupling slot. This configuration reshapes surface currents and improves the radiation performance across a broad frequency range. The design offers significant advantages such as reduced antenna size, minimized influence of higher-order modes, and maintained low cross-polarization (XP) level. Experimental results demonstrate that the proposed metasurface-based slot antenna provides a bandwidth of 29.6% (23–31 GHz) with a return loss better than 10 dB. It achieves a peak gain of 9.43 dB and exhibits an XP level below − 26 dB and − 48 dB at $\varphi = 0^{ \circ }$ and $\varphi = 90^{ \circ }$ planes, respectively. The physical dimensions of the antenna are 0.9λ_0_ × 0.9λ_0_ × 0.08λ_0_, where λ_0_ is the free space wavelength at 27 GHz, resulting in an approximately 41% reduction compared to the conventional metasurface patch antenna. Moreover, the design proves to be well-suited for MIMO systems, enabling close placement of antenna elements without degrading their radiation patterns. The experimental results in 1 × 2 and 2 × 2 MIMO configurations represent that the isolation between antenna elements are better than 18 dB and 21 dB, respectively. The performance of the antennas remains stable in both configurations, effectively addressing concerns such as beam squint and eliminating the common issue of beam splitting observed in conventional metasurface MIMO antennas. Moreover, the envelope correlation coefficient value in both MIMO configurations is lower than 0.003. This significant advancement offers a promising solution for compact 5G mm-wave massive MIMO applications.

## Introduction

The demand for high speed and small latency has led to the widespread adoption of 5G wireless communication systems across various applications, including Internet of Things (IoT), medical devices, handheld devices, and virtual reality^1^. To accommodate these applications, the Federal Communications Commission (FCC) has recommended the utilization of multiple mm-wave frequency bands, such as 24 GHz/28 GHz/37 GHz/39 GHz, and 60 GHz, for 5G deployments^2^. The mm-wave band is highly desirable due to its ability to facilitate high-speed data transfer, making it a popular choice for antenna designs^3^. However, path loss poses a significant challenge in the 5G mm-wave band, necessitating the use of high-gain antennas^4,5^. In this regard, metasurface antennas offer an efficient and effective solution to enhance gain and radiation capabilities while simultaneously reducing size and broadening bandwidth compared to conventional antennas. Different types of the coupling slot antenna, such as rectangular^6–8^, loop^9^, circular^10^, and I-shaped^11^, has been employed in metasurface antenna designs.

The application of the CMA in the design of antennas for 5G communication systems has been explored in previous studies^12–16^. However, some of these antennas encountered challenges related to unwanted higher-order modes within the desired frequency band. These modes adversely affected the antenna's performance by reducing gain^13^, increasing cross-polarization (XP) level^17^, and introducing irregularities in radiation patterns in MIMO configurations^18^. To overcome these challenges, researchers have investigated two methods: mode shifting and mode repolarizing^18,19^. In^13^, three rectangular strips were introduced between the main H-shaped structure to suppress modes 2 and 3 and enhance antenna gain. However, this resulted in increased antenna dimensions and reduced bandwidth. In^15^, a mode suppressor comprising an array of square patches was used in each corner of the main antenna to suppress modes 3 and 4. However, this approach led to significant increase in the overall dimensions of the antenna. In^17^, a modified patch metasurface structure with a 4 × 4-element array was designed to reduce XP levels by shifting the strongest surface current distributions from the central patches to the side patches. This was achieved by increasing the dimensions of the side patches, which in turn increased the antenna overall size. Additionally, this structure required two differential excitation ports for each mode, which further complicated the design and could lead to increased XP level. Another approach suggested in^18^ utilized metal pins and slots on metasurface patches to suppress undesired higher-order modes. However, the inclusion of metal pins added complexity to the antenna structure. Overall, the current methods employed to suppress higher-order modes have resulted in complex antenna structures with larger dimensions. This makes them less suitable for modern compact massive MIMO systems, where space is a constraint especially in the mm-wave band.

In 5G wireless communication systems, MIMO configurations are widely used to enhance system capacity and enable simultaneous connections for multiple users, leading to higher transmission rates^20^. However, in modern compact systems with closely spaced antenna elements and proximity to the transceiver systems, mutual coupling between elements becomes a common issue, resulting in a decrease in overall antenna performance^21–34^. Several decoupling techniques have been explored to address this challenge, including Defected Ground Structures (DGS)^22–24^, electromagnetic band gap (EBG) structures^25–27^, and self-decoupling antennas^28,29^. For instance, the implementation of slits in the radiators of two radiating elements has achieved high isolation among antenna radiators^30^. In^35^, CMA is employed to design a DGS for a four-element patch MIMO antenna, which effectively reduces mutual coupling between the elements. However, this design suffers from a relatively high beam squint angle. Similarly,^36^ proposes a 2 × 2 non-uniform metasurface to enhance the radiation characteristics of a MIMO antenna, but this approach results in beam splitting and exhibits a high beam squint angle away from the desired broadside direction. Another metasurface-based MIMO antenna design presented in^37^ exhibits a beam squint angle ranging from − 16° to 25°. Despite their effectiveness, some of these methods introduce complexities and increase dimensions of the MIMO structure. Additionally, they fail to preserve the radiation patterns of the individual elements, leading to distortion in multi-port systems. Therefore, the challenge lies in designing mm-wave MIMO antennas that achieve high isolation between elements without requiring additional spacing, while also preserving the desired radiation patterns.

In this paper, we present an approach for designing metasurface antennas that effectively suppresses higher-order modes, making them suitable for MIMO configurations. The design methodology utilizes the CMA method and takes a metasurface antenna with a 3 × 3 array of symmetric square patches as a reference. In this conventional metasurface, the first two modes are degenerate, and higher-order modes exist within the desired frequency band, necessitating mitigation. To address this, the proposed design incorporates two rectangular slits on both the right and left sides of each patch. These slits effectively eliminate the degeneracy between the first two modes. Furthermore, the surface currents associated with the higher-order modes are redirected from the central patch to the side patches, thereby enhancing the radiation of the first (fundamental) mode. To further enhance performance, two rectangular stubs are added on the other sides of the patches. This straightforward approach not only reduces the dimensions of the antenna but also significantly reduces cross-polarization (XP) level. To validate the performance of the proposed antenna design, 1 × 2 and 2 × 2 MIMO configurations are analyzed, without requiring additional spacing between the elements. The results demonstrate stable radiation patterns and promising overall performance. This approach provides a simple solution for addressing pattern distortion in multi-port systems while maintaining high isolation and preserving radiation patterns.

The paper is structured as follows: in “Introduction” section introduces the importance of metasurface design in antenna engineering, emphasizing its potential applications in 5G and beyond. In “Design method” section, the metasurface design methods and procedures are thoroughly explained, covering underlying principles, theories, and simulation tools used. In “Feeding the proposed metasurface by a coupling aperture” section presents a comprehensive analysis of the proposed metasurface antenna's performance, including radiation characteristics, gain, and impedance matching, with comparisons to conventional designs. In “Evaluation of the proposed antenna performance in MIMO configurations” section, the evaluation of the metasurface antenna in 1 × 2 and 2 × 2 MIMO configurations is discussed, considering envelope correlation coefficient (ECC) and diversity gain (DG). Finally, in “Conclusion” provides a concise conclusion summarizing the key findings.

## Design method

In the following, we will discuss the implementation of the CMA method, followed by the definition of the modal significance (MS) parameter and the mode manipulation method.

### Characteristic mode analysis (CMA)

#### Maximizing fundamental mode excitation

The CMA method is a widely utilized technique for antenna design, particularly in the context of mode manipulation. It enables the determination of crucial antenna characteristics, such as operating frequency, radiation patterns, and surface currents for various modes^38–41^. The CMA approach relies on the eigenvalue equation, which can be expressed mathematically as^38^:

1 $$\left[ X \right]J_{n} = \lambda_{n} \left[ R \right]J_{n}$$

where $\left[ R \right]$ and $\left[ X \right]$, respectively, represent the real and imaginary parts of the impedance matrix in the method of moment (MOM) analysis. $\lambda_{n}$ is the eigenvalue, which varies within $- \infty < \lambda_{n} < + \infty$, and corresponds to the eigencurrent of the *n*th mode, $J_{n}$. According to the CMA approach, the total current on a perfectly electrically conducting (PEC) structure can be expressed as a linear combination of the currents associated with all the modes present. This can be written as^41^:

2 $$J = \sum\limits_{n} {\alpha_{n} J_{n} }$$

where $\alpha_{n}$ is modal weighting coefficient for each mode and represents the contribution of each mode to the total radiated power. This parameter can be defined as follow^41^.

3 $$\alpha_{n} = \frac{{V_{n}^{i} }}{{1 + j\lambda_{n} }}$$

where $V_{n}^{i}$ is modal-excitation coefficient and can be calculated from Eq. (4)^41^.

4 $$V_{n}^{i} = \left\langle {J_{n} ,E^{i} } \right\rangle = \mathop{{\int\!\!\!\!\!\int}\mkern-21mu \bigcirc}\limits_{s} {J_{n} \cdot E^{i} ds}$$

The mode excitation parameter $V_{n}^{i}$, plays a crucial role in antenna design and is defined as the inner product of the surface current of the *n*th mode and the applied electric field. This parameter signifies that for the effective excitation of each mode, the excitation source ($E^{i}$) need to be positioned where the modal surface current of that mode is strongest^17^. However, this may lead to the excitation of unwanted modes, which can negatively affect the antenna performance and should be addressed in our design.

#### Quantifying the significance of each mode excitation

Here we use the modal significance (MS) of each mode as another crucial parameter in antenna design^38^. According to^39,40^, a mode can be excited if its MS at a particular frequency *f* is greater than 0.707. MS can be calculated as follows^38^:

5 $${\text{MS}}_{n} = \frac{1}{{\left| {1 + j\lambda_{n} } \right|}}$$

For example, when the MS value for a mode is equal to 1, the eigenvalue $\lambda_{n}$ becomes zero.

#### Higher order mode manipulation

Typically, unwanted modes can be shifted to higher frequencies, but this has the drawback of potentially increasing the size of the metasurface^13^. Moreover, this approach may not effectively shift the majority of the unwanted modes to higher frequencies, as desired^18^. Consequently, the most effective strategy to mitigate the influence of higher-order modes revolves around modifying the distribution of surface currents of these modes. The aim here is to minimize the surface currents of unwanted higher-order modes while simultaneously maximizing the surface currents associated with the fundamental mode in the metasurface patches. Alternatively, it is advantageous if the direction of the surface currents of unwanted modes is orthogonal to that of the fundamental mode or moves to the side patches in opposite directions to each other. This ensures that the undesired modes are not simultaneously excited with the fundamental mode. In this case, the $J_{n}$ value decreases and, in turn, reduces the excitation coefficient of the higher-order mode, i.e. $V_{n}^{i}$, These considerations are crucial for minimizing the impact of unwanted modes on the XP level and enhancing the performance of the antenna.

### Design procedure of the novel metasurface

**Figure 1:**
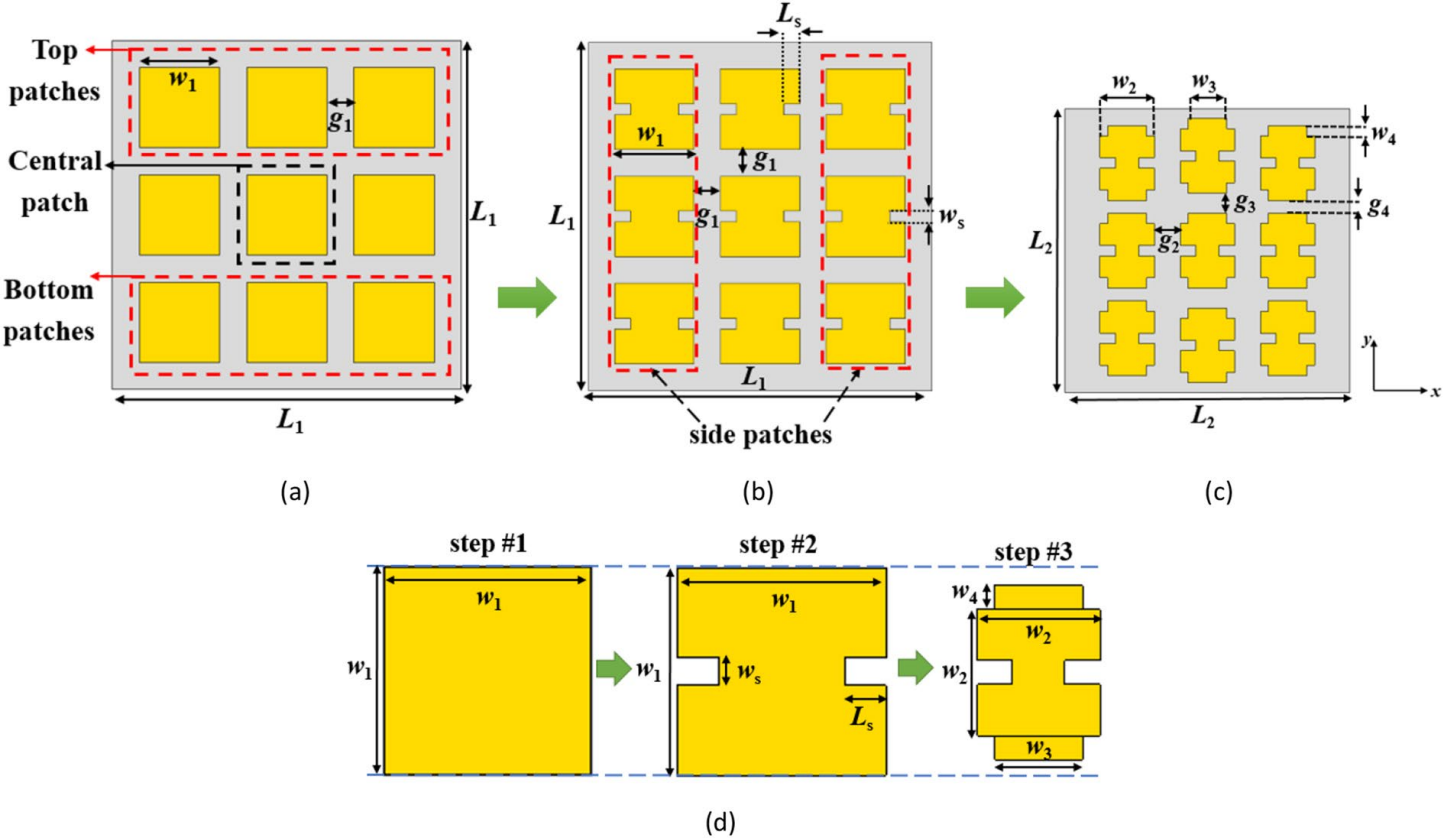
Evaluation process of the proposed metasurface. (**a**) conventional metasurface (step #1), (**b**) modified metasurface (step #2), (**c**) proposed final metasurface with reduced dimensions (step #3), (**d**) evolution of the unit cells in each step.

**Figure 2:**
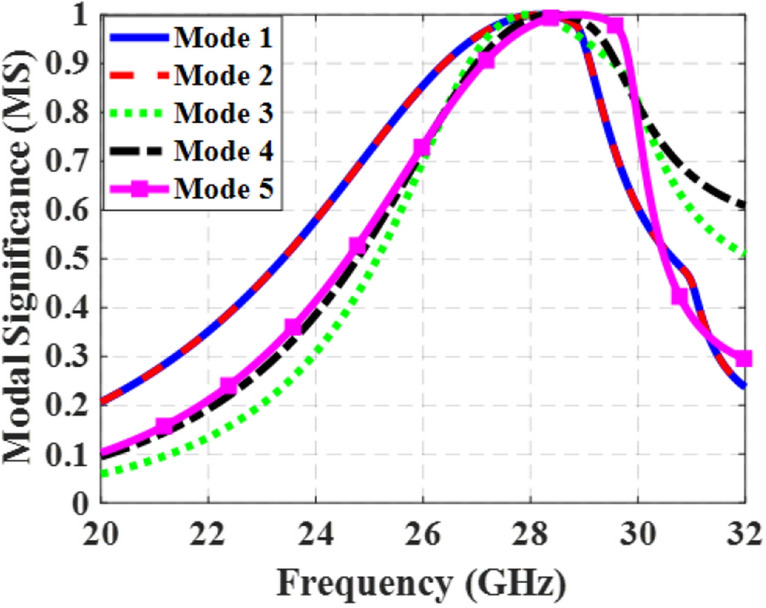
MS curves of the conventional metasurface (step #1) for the first five modes.

In order to assess the design steps of the proposed metasurface, Fig. 1 presents a visual representation of the design method. To clarify the design approach, an initial analysis is carried out on a configuration of square patches metasurface, as displayed in Fig. 1a. This configuration consists of a 3 × 3 array of square patches printed on an RT/duroid 5880 substrate with a thickness of 0.787 mm, *ε*_*r*_ = 2.2, and tan*δ* = 0.0009. The MS curves of the first five modes of this structure are obtained using the CMA calculation in CST STUDIO SUITE® software, as depicted in Fig. 2. Due to the symmetrical nature of this structure, the MS curves reveal that first and second modes are degenerate, while the MS values for the higher-order modes (3–5) exceed 0.707 within the desired frequency range of 24.5–29.5 GHz. The existence of these higher modes has a detrimental effect on the performance of the fundamental mode.

**Figure 3:**
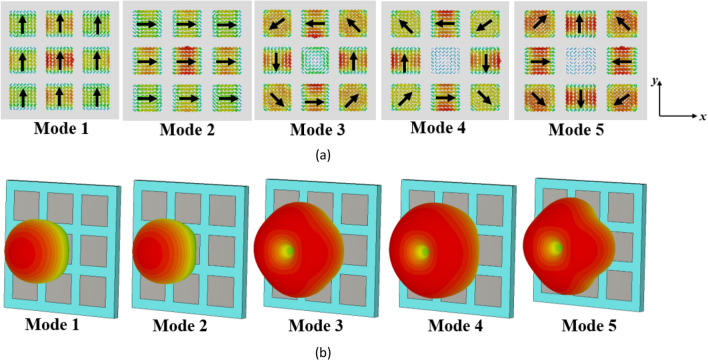
The evaluation results of the conventional metasurface (step #1). (**a**) modal currents, (**b**) directivity patterns of the first five modes at 27 GHz.

For the first five modes of the conventional metasurface, the surface currents and radiation patterns at 27 GHz are shown in Fig. 3a and b, respectively. As can be seen, the surface currents of modes 1 and 2 are identical but rotated by 90° (orthogonal to each other), and they are strong on the central patch. In contrast, modes 3, 4, and 5 have minimal current on the central patch and display nulls in the broadside direction in their far-field patterns, making them undesirable modes. It can also be observed that mode 1 can be excited by supplying power to the central patch where the surface current is strongest based on Eq. (4).

**Figure 4:**
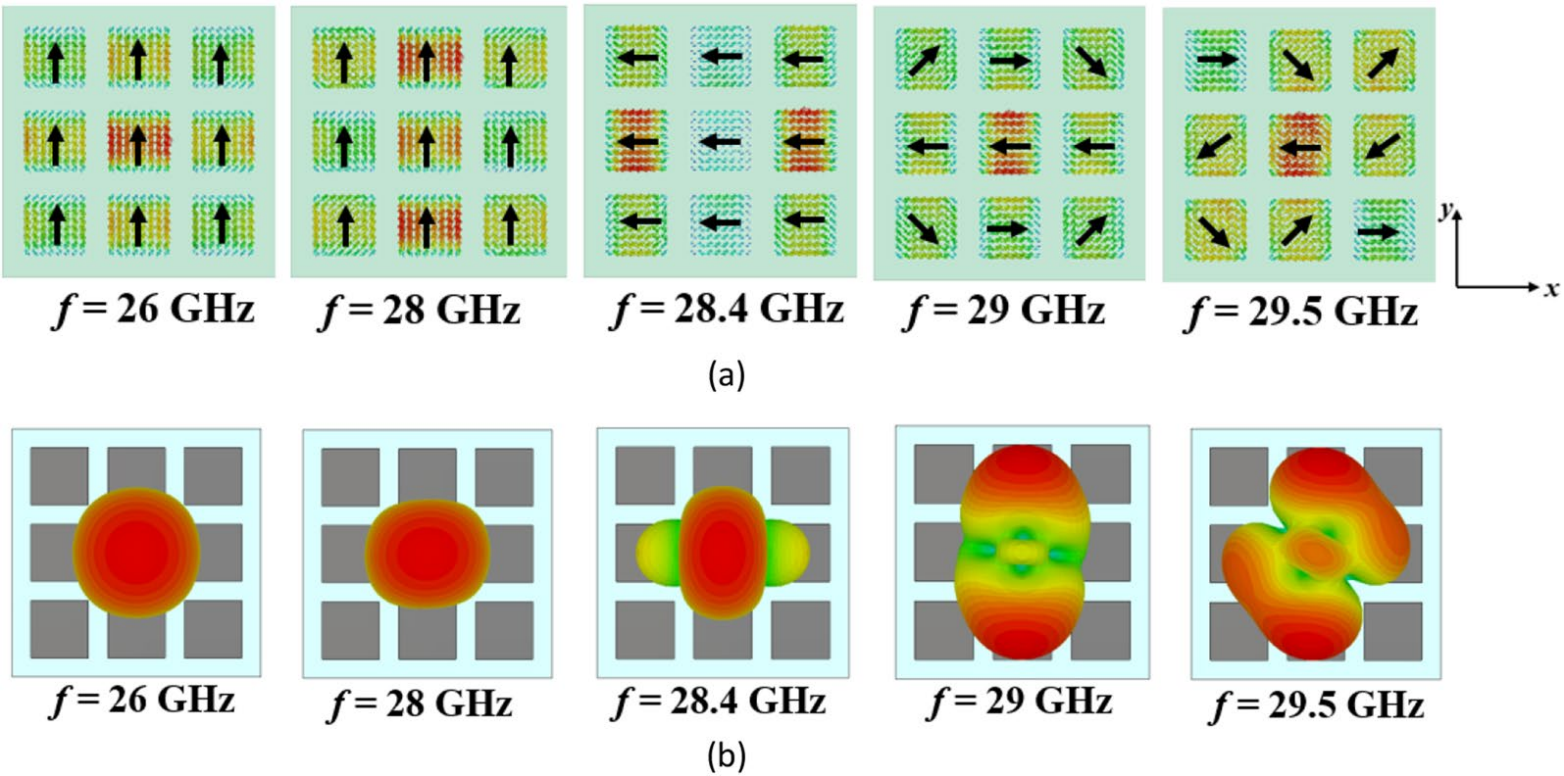
(**a**) Surface currents and (**b**) directivity patterns of mode 1 in the conventional metasurface (Step #1) at different frequencies.

However, the surface currents and radiation patterns of the modes exhibit frequency-dependent behavior. Hence, we need to further analyze the performance of mode 1 over the desired frequency range. Figure 4 presents its surface currents and directivity patterns at various frequencies. For frequencies below 28.4 GHz, the surface currents align in the same direction, resulting in a broadside radiation pattern. However, at 28.4 GHz, the currents become orthogonal to those at lower frequencies, causing a radiation with orthogonal polarization. As we move from 29 to 29.5 GHz, the surface currents on the patches no longer align in the same direction. As can be seen, the presence of unwanted higher-order modes results in changes in the distribution of surface currents, leading to degradation in the radiation patterns of the antenna at higher frequencies. As a result, at these frequencies, mode 1 cannot be efficiently excited by a coupling rectangular slot beneath the central patch; instead, mode 2 is excited due to the degeneracy and orthogonality between the two modes. This renders the conventional metasurface unsuitable for broadside radiation at higher frequencies, limiting its use for the wideband 5G frequency range (24.5–29.5 GHz).

When modifying the surface currents of unwanted higher-order modes, the impact on the fundamental mode (mode 1) should be considered. Based on the observations in Figs. 3a and 4, it can be concluded that the surface currents of mode 1 are consistently *y*-polarized on all patches, while that of mode 2 is *x*-polarized at frequencies below 28.4 GHz. However, this behavior changes for both modes at 28.4 GHz. Therefore, the key objective of the modification in Step #2 have three main purposes: (1) creating an asymmetric structure to eliminate the degeneracy between the first and second modes, (2) maintaining the orientation of surface currents of the fundamental mode (first mode) below 28.4 GHz, and (3) rotating the surface currents of the first mode at frequencies beyond 28.4 GHz to align them as closely as possible with currents at lower frequencies within the desired range. Additionally, the surface currents of higher-order modes must be relocated from the central patch to the surrounding patches or adjusted to be orthogonal to that of mode 1. By achieving these objectives, the metasurface can be optimized to enhance the performance of mode 1 while mitigating the influence of unwanted higher-order modes. This will result in a broadside far-field radiation pattern over the desired wide frequency range (24.5–29.5 GHz). However, it is also important to consider the impact of reshaping the surface currents of mode 1 on undesired higher-order modes.

**Figure 5:**
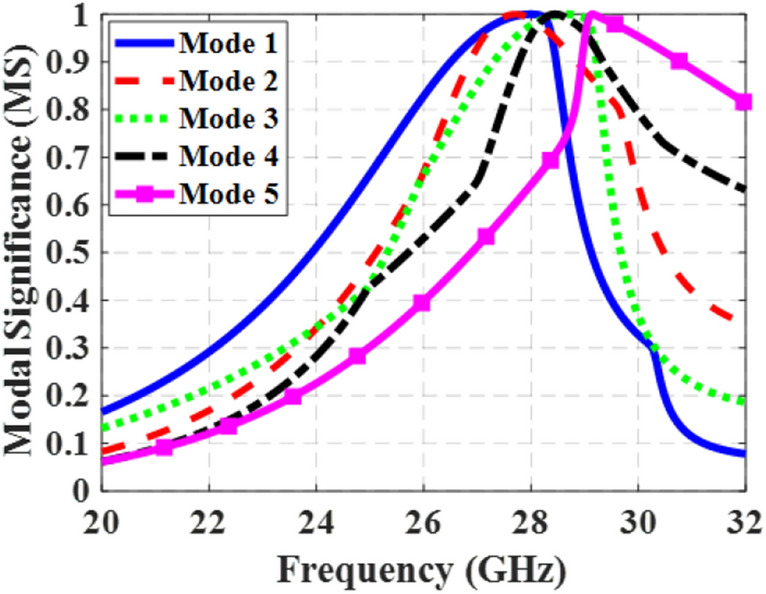
MS curves of the modified metasurface (step #2) for the first five modes.

To address the aforementioned challenges, adjustments were made in design Step #2 by introducing rectangular slits on both sides of all 9 patches. This is shown in Fig. 1b. The slits were oriented in the *x*-direction to maintain the *y*-polarized surface currents of mode 1 at frequencies below 28.4 GHz. Figure 5 illustrates the MS curves for the first five modes of the modified structure. The slits effectively lowered the resonant frequency of mode 1 and eliminated the degeneracy between mode 1 and mode 2 by increasing the electrical length of the *y*-polarized surface currents. Now, the bandwidth of mode 1, where MS ≥ 0.707, spans from 25.2 to 28.72 GHz, while that of mode 2 ranges from 26.1 to 29.87 GHz. However, higher-order modes still exist within the desired frequency band, and mode 1 still does not cover the desired frequency range.

It is important to note that, as mentioned in^42^, the MS value alone is not sufficient for radiation. The direction of the surface currents, as well as the value and location of the applied feeding EM fields, also significantly influence the overall performance of the metasurface antenna.

**Figure 6:**
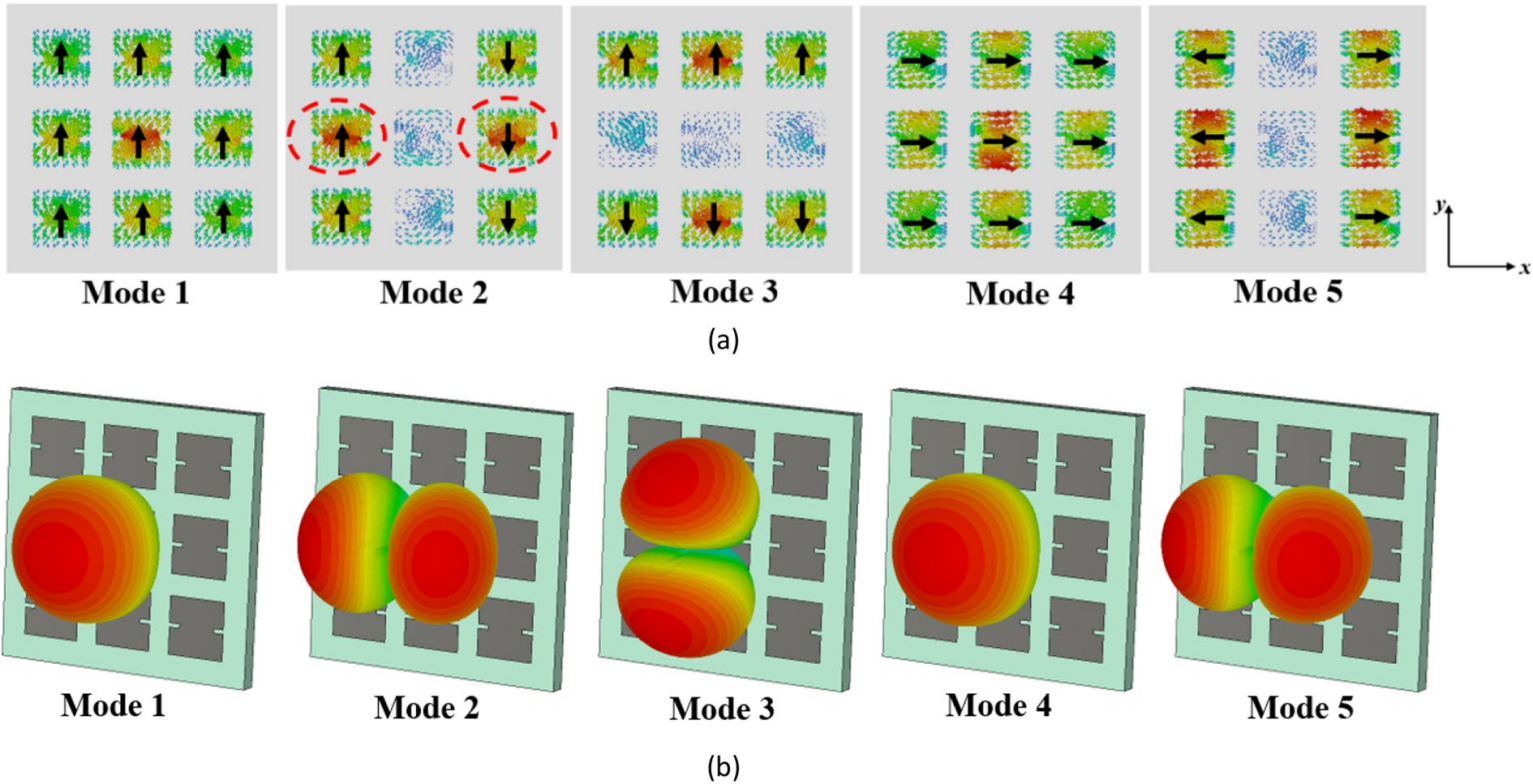
The results of the modified metasurface in Step #2. (**a**) modal currents, and (**b**) directivity patterns of the first five modes at 27 GHz.

Figure 6 presents the surface currents and directivity patterns of the first five modes in the modified configuration (step #2) at 27 GHz. The surface current of mode 1 remains unchanged, while a significant portion of the surface currents of mode 3 has shifted to the top and bottom patches, with maximum values no longer located on the central patch. Specifically, the surface currents of mode 4 are in the + *x* direction across all patches, while those of mode 5 are in both + *x* and − *x* directions on the side patches. These surface currents of modes 4 and 5 are orthogonal to those of mode 1 and are not excited with mode 1 when fed by a rectangular coupling slot at the bottom of the central patch. The maximum surface currents of mode 3 have been relocated to the upper and lower side patches, resulting in a significant reduction in the surface currents on the central patch. As $J_{3}$ decreases, $V_{3}^{i}$ will decrease according to (4). Therefore, the influence of mode 3 on the performance of mode 1 becomes negligible. Additionally, to excite mode 2 effectively, two coupling slots with differential phase feeding are required beneath the patches marked in Fig. 6a. A single coupling slot is insufficient. These findings highlight the advantages of using slits on the sides of patches as a simple yet efficient approach. However, further reduction of the surface currents of modes 2 and 3 on the central patch is preferred as they are not radiating at broadside.

**Figure 7:**
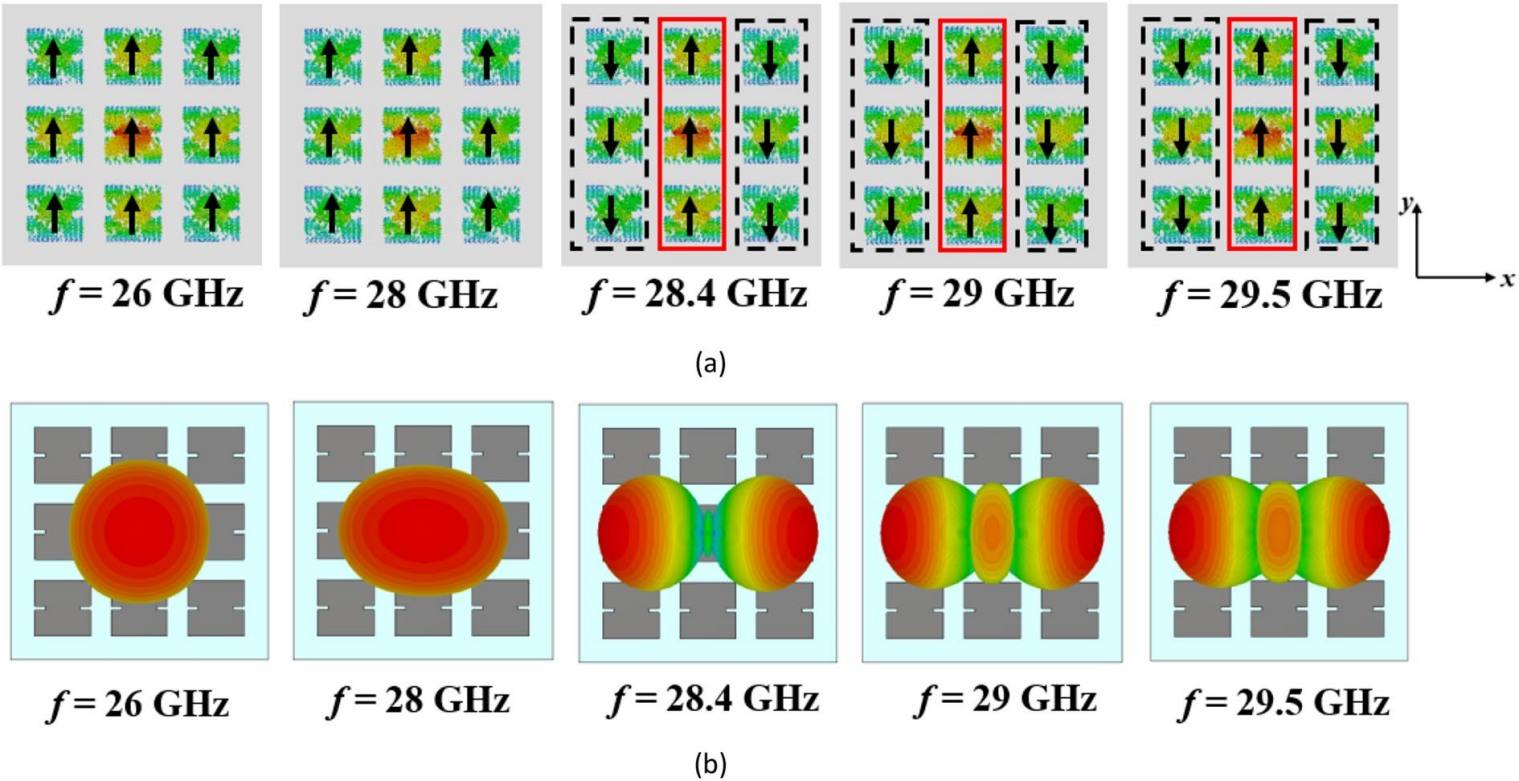
(**a**) Surface currents and (**b**) directivity patterns of mode 1 in the modified metasurface (Step #2) at different frequencies.

After step #2, we are still not satisfied as we have a narrow bandwidth. To illustrate, Fig. 7 shows the surface currents and radiation patterns of mode 1 in the modified metasurface (Step #2) at different frequencies. After introducing the slits, Fig. 7a demonstrates consistent surface currents at frequencies below 28.4 GHz. Furthermore, between 28.4 and 29.5 GHz, a significant positive change in the direction of surface currents is observed. However, the currents on the side patches flow in the opposite direction compared to the central patches. Consequently, these surface currents cancel each other out within this frequency range, resulting in radiation patterns that are not in the broadside direction. Therefore, further modifications are still required to increase the MS values of mode 1, as well as reshape the surface currents from 28.4 to 29.5 GHz to be in the same direction on all patches. Additionally, further reduction of the surface currents of modes 2 and 3 on the central patch is necessary to eliminate their effects on the fundamental mode radiation. These findings indicate that there is still potential to optimize the structure for operation over the desired broad frequency range while maintaining stable and broadside radiation patterns.

**Figure 8:**
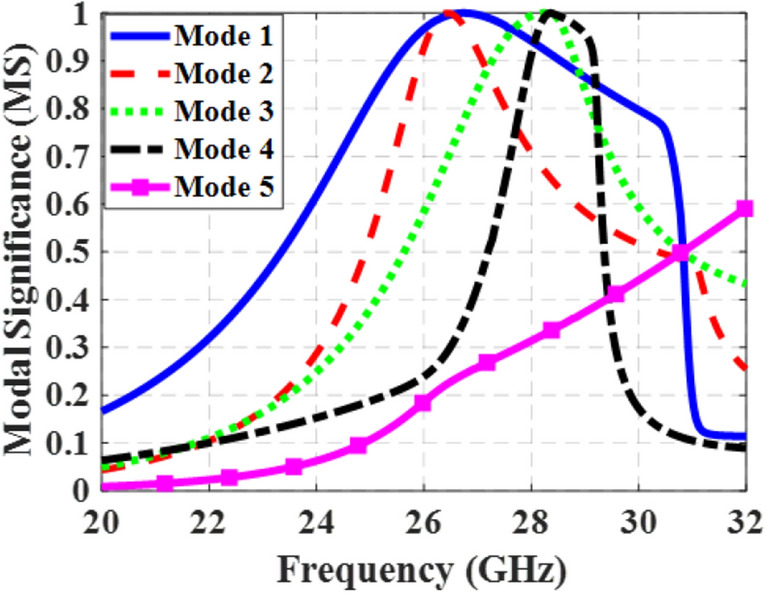
MS curves of the proposed metasurface (step #3) for the first five modes.

Step #3: In order to improve the performance of the metasurface and ensure consistent operation of mode 1 across the desired frequency range, further modifications are implemented. By incorporating two extra stubs at the top and bottom of the patches in the *y*-direction, the second structure is transformed into Step #3, as depicted in Fig. 1c. This modification serves to minimize the impact of higher-order modes on the performance of the first mode. Consequently, the alignment of surface currents of mode 1 remains consistent at lower frequencies but rotate at higher frequencies (from 28.4 to 29.5 GHz) to align with the currents at lower frequencies (*y*-oriented). Figure 8 illustrates the MS curves for the first five modes of the proposed design, indicating that the MS of mode 1 remains above 0.707 throughout the desired frequency range.

**Figure 9:**
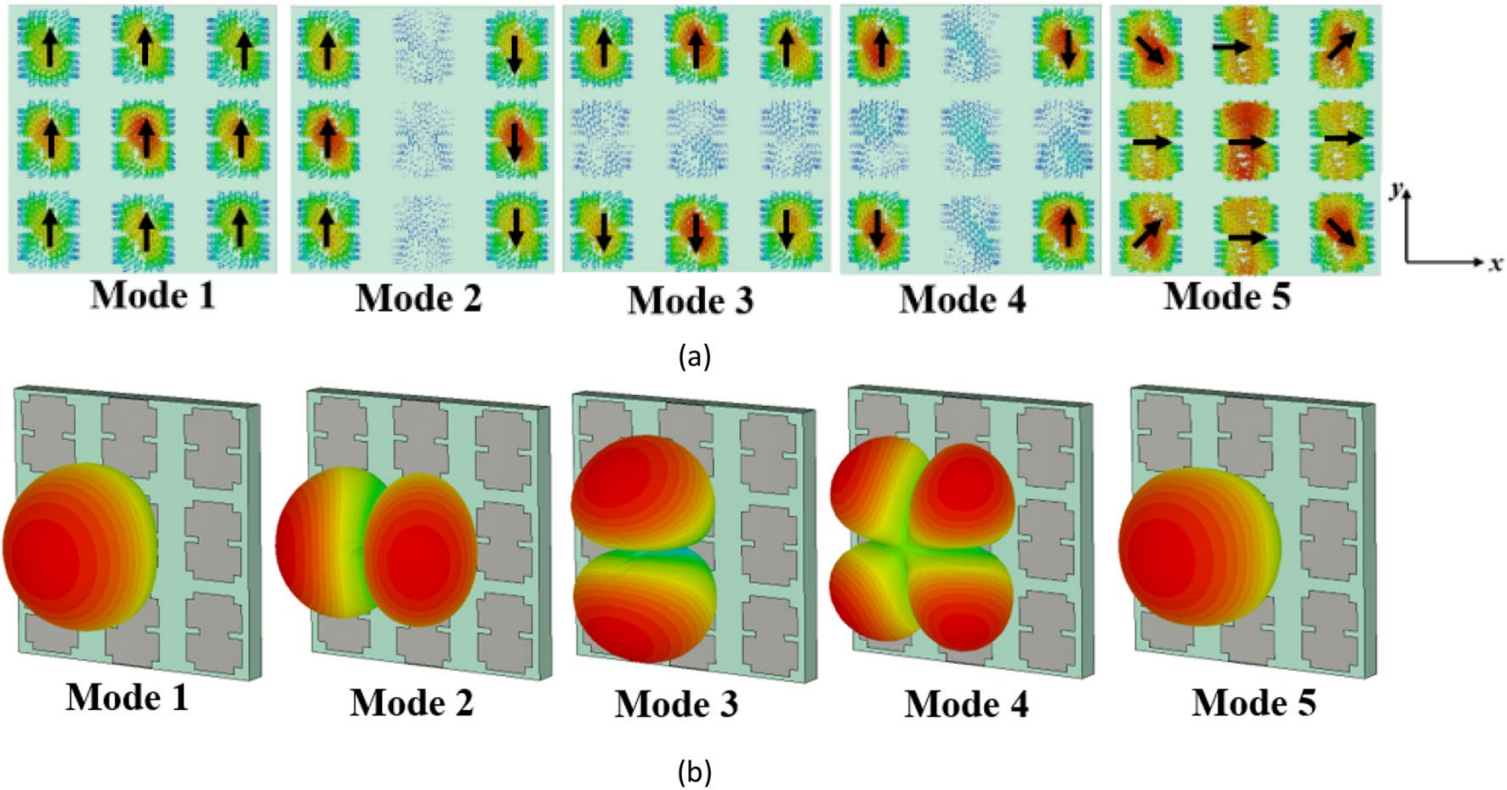
(**a**) Modal currents, (**b**) directivity patterns of the first five modes in the proposed metasurface (Step #3) at 27 GHz.

**Figure 10:**
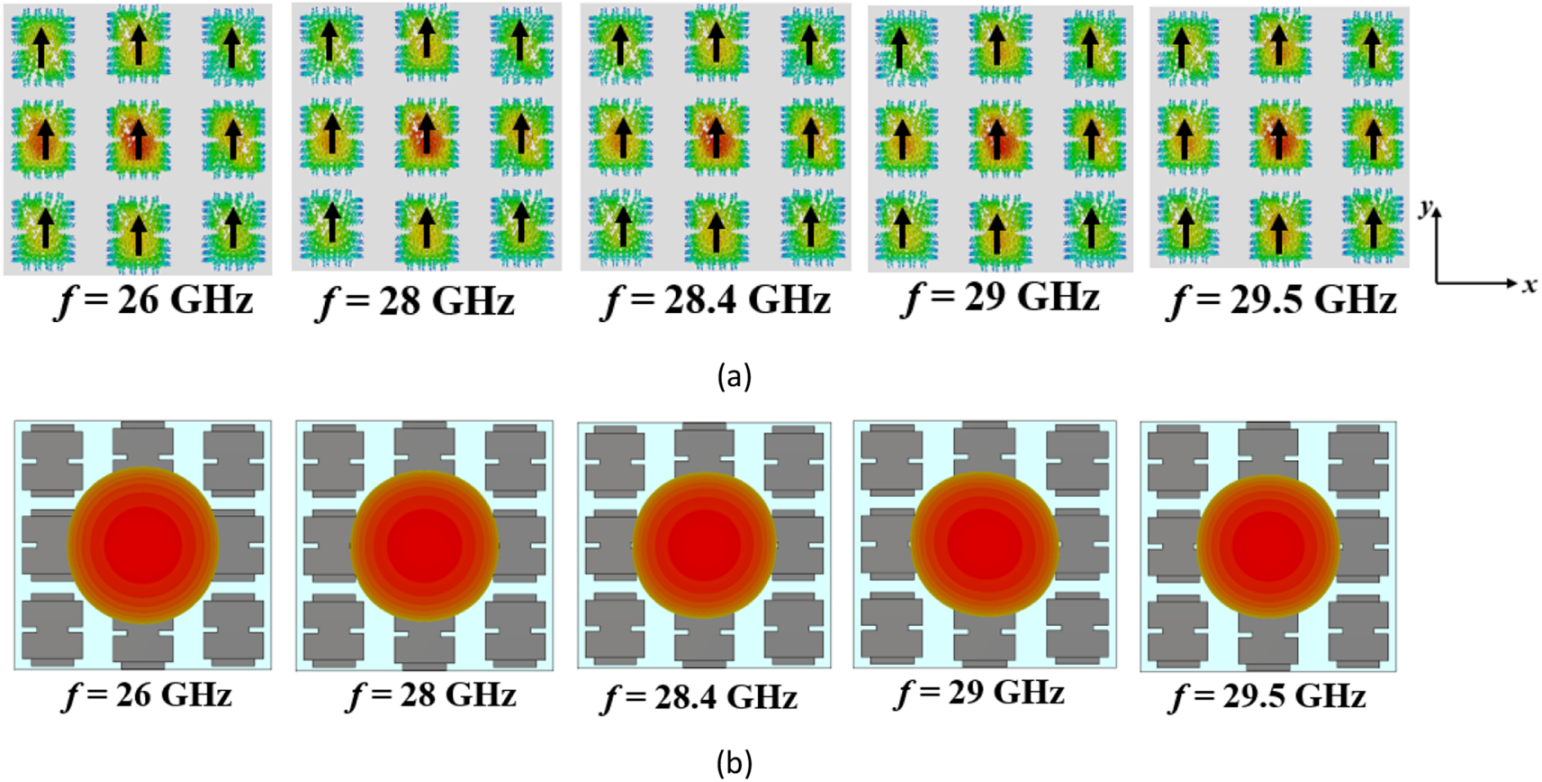
(**a**) Surface currents and (**b**) directivity patterns of mode 1 in the proposed metasurface (Step #3) at different frequencies.

Figure 9 displays the surface currents and radiation patterns of the first five modes in the proposed metasurface at 27 GHz, while Fig. 10 illustrates the results of mode 1 at different frequencies. Mode 1 consistently exhibits a broadside radiation pattern throughout its operational frequency range. On the other hand, Mode 5, has an MS value below 0.707 in the desired frequency band and is shifted to higher frequencies. Moreover, the surface currents of mode 5 are orthogonal to those of mode 1, making it impossible to excite with mode 1. Modes 2–4 display reduced currents on the central patch. Consequently, even though these modes surpass an MS value of 0.707 within specific frequencies in the desired range, the currents in the side patches flow in opposite directions. To excite these modes, multiple coupling slots with differential phase feeding are required. As a result, these modes cannot be excited with a single rectangular coupling slot under the central patch like mode 1. By modifying the direction of surface currents of mode 1 in the frequencies between 28.4 and 29.5 GHz, eliminating the degeneracy between modes 1 and 2, and relocating the maximum currents of higher-order modes from the central patch to the surrounding patches, the orientation and density of surface currents remained consistent, resulting in stable radiation patterns across the entire operating band and potential distortions in the radiation patterns of MIMO configurations can be effectively resolved.

Additionally, incorporating slits and stubs by extending the path length of surface currents has resulted in a reduction of approximately 41% in the occupied area of the metasurface compared to the conventional design, shrinking it from 1.17λ_0_ × 1.17λ_0_ to 0.9λ_0_ × 0.9λ_0_ (λ_0_ is the free space wavelength at 27 GHz). Table 1 provides the optimized parameters for the metasurfaces.

**Table 1:** Dimensions of the metasurfaces (unit: mm).

*w*_1_	*w*_2_	*w*_3_	*w*_4_	*w*_s_	*L*_s_	*g*_1_	*g*_2_	*g*_3_	*g*_4_	*L*_1_	*L*_2_
3	2	1.5	0.2	0.3	0.4	1	0.9	0.7	0.5	13	10

## Feeding the proposed metasurface by a coupling aperture

**Figure 11:**
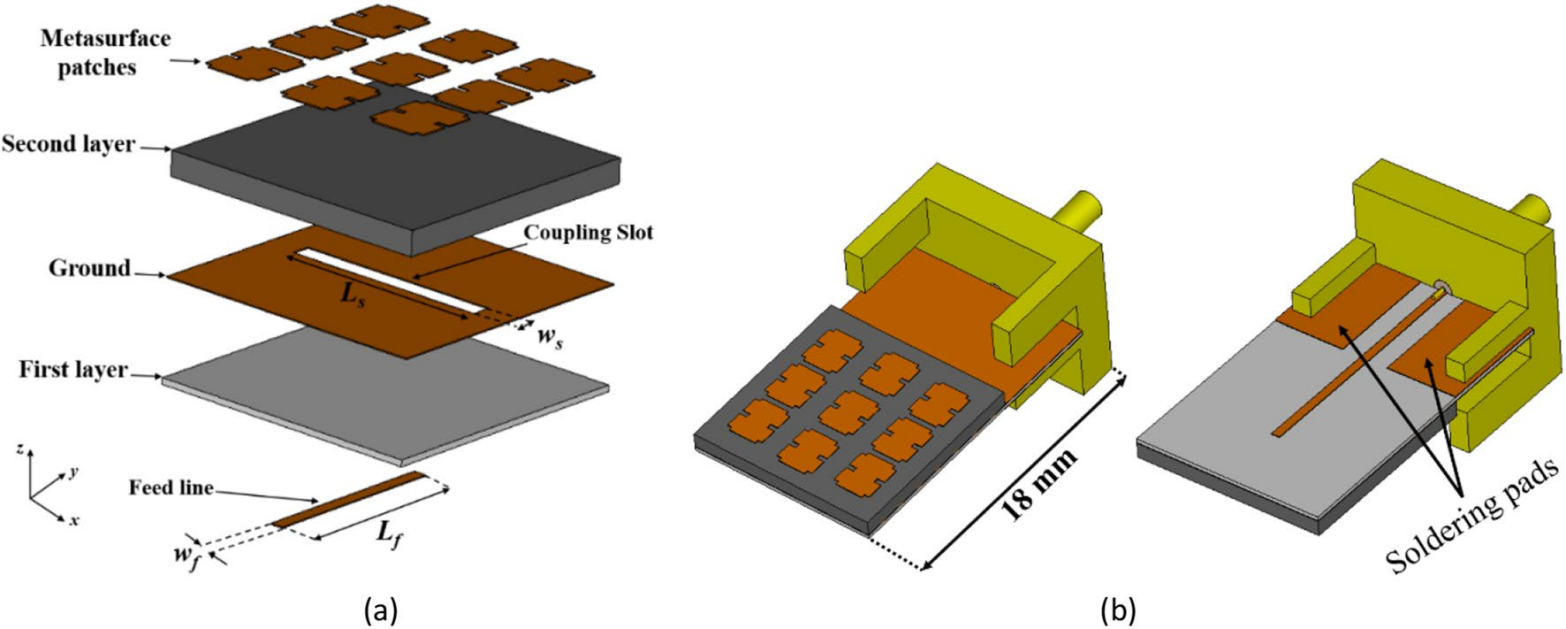
The structure of the proposed metasurface antenna. (**a**) 3D layout, (**b**) top and bottom views of the simulated antenna with an RF connector and soldering pads. (*w*_*f*_ = 0.42 mm, *L*_*f*_ = 6.5 mm, *w*_*s*_ = 0.65 mm, *L*_*s*_ = 6.7 mm).

The configuration of the proposed two-layer metasurface antenna is depicted in Fig. 11a. The top metal of the first layer acts as the ground plane consists of a coupling slot at the center that is etched on an RO4003C substrate with a thickness of 0.203 mm, *ε*_*r*_ = 3.38, and tan*δ* = 0.0027. A microstrip line is located at the bottom to feed the coupling slot. The second layer comprises the proposed metasurface, which is printed on an RT/duroid 5880 substrate with a thickness of 0.787 mm, *ε*_*r*_ = 2.2, and tan*δ* = 0.0009. Due to the small size of the antenna, there is limited space available for soldering the connector onto it. As a solution, the length of the feed layer, particularly the ground plane, first layer and feed line has been extended to make room for the connector, as illustrated in Fig. 11b. Additionally, two metal pads have been incorporated on the backside for soldering of the connector leads.

**Figure 12:**
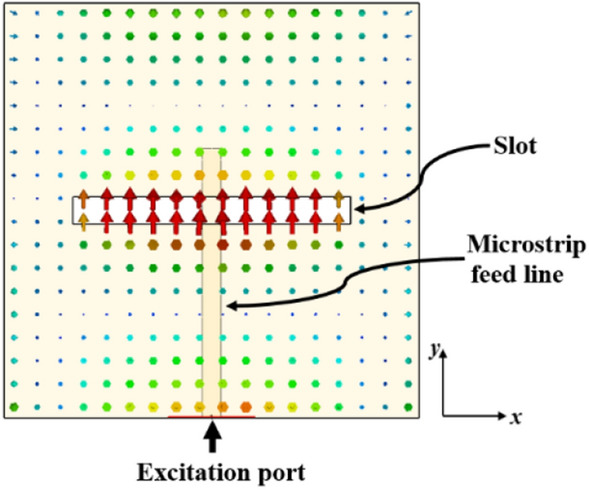
The distribution of the electric field on the slot antenna without the proposed metasurface layer at 27 GHz.

The antenna design incorporates the use of the indirect feeding method, i.e. proximity coupling, to feed the metasurface patch antennas with a microstrip line. To prevent undesired radiation from the feed line which has an adverse effect on the antenna performance, a thin substrate with a high dielectric constant is chosen for the feed line. Conversely, a thicker substrate with a lower dielectric constant is used for the metasurface antenna to enhance radiated power, bandwidth, and overall efficiency. Figure 12 illustrates the distribution of the electric field on the slot antenna without the proposed metasurface layer at 27 GHz. The E-field within the slot is uniform and oriented in the *y*-direction. According to the modal-excitation coefficient ($V_{n}^{i}$) from Eq. (4), by positioning the coupling slot in the middle of the proposed metasurface, where the surface currents of mode 1 are strongest, it guarantees the proper excitation of mode 1.

**Figure 13:**
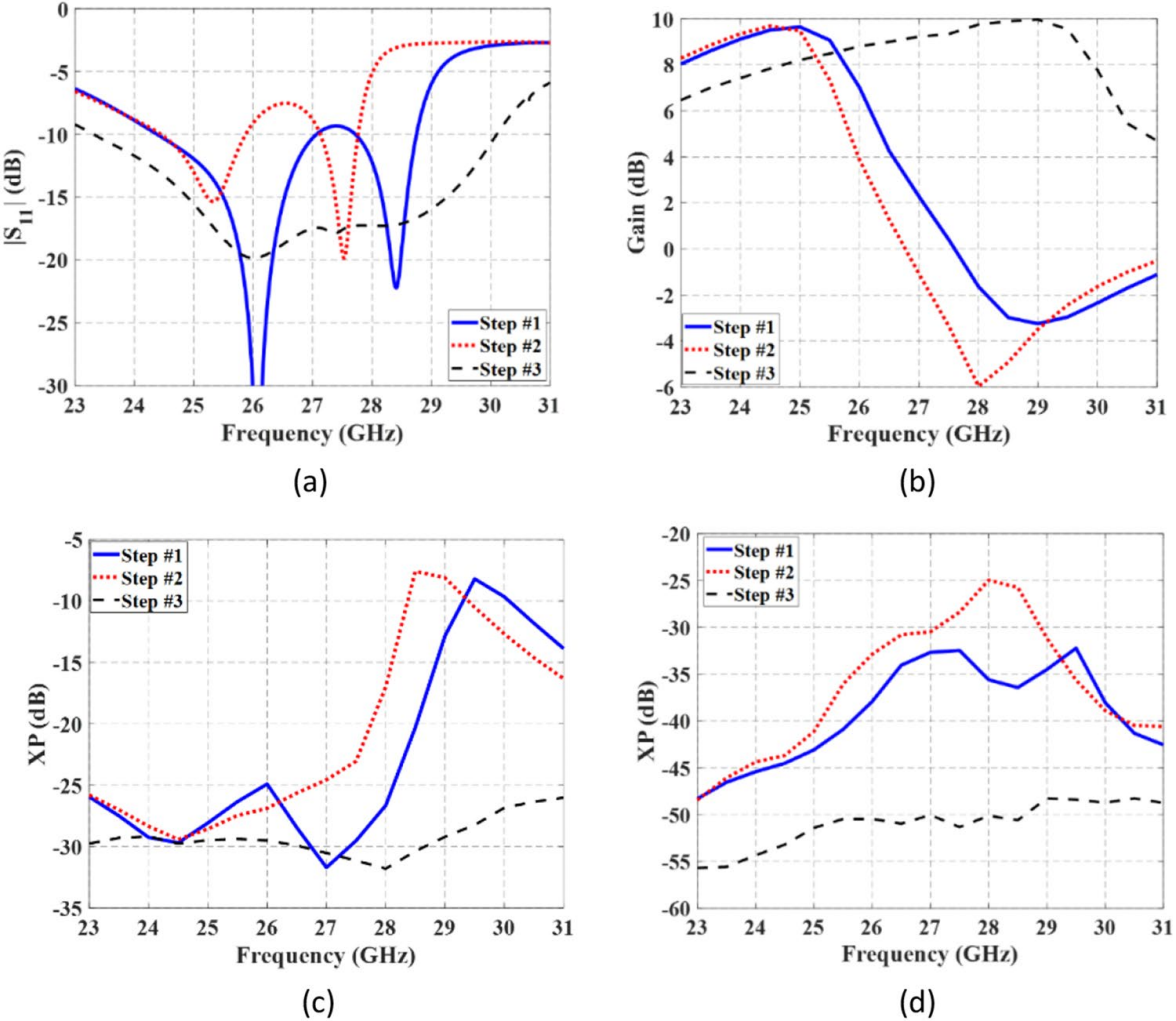
Comparison the simulated results of three metasurface-based slot antennas. (**a**) |S_11_|, (**b**) variation of the gain at broadside direction; variation of the XP level at (**c**) $\varphi = 0^{ \circ }$ and (**d**) $\varphi = 90^{ \circ }$ planes.

Figure 13 presents a comparison between the simulated results of metasurface antennas using three investigated metasurfaces, as depicted in the steps of Fig. 1. In Fig. 13a, |S_11_| for the three metasurface-based slot antennas are illustrated. As can be seen, the proposed design in Step #3 covers a frequency range of 23.3–30.1 GHz. The variation of gain with frequency in the broadside direction is also presented in Fig. 13b. Comparatively, the dimensions of the structure in Step #3 are smaller than the other two, leading to lower gain at frequencies below 25 GHz. However, at higher frequencies, the first two steps suffer from the presence of higher-order modes and misalignment of surface currents, resulting in radiation patterns deviating from the broadside direction and a significant drop in gain.

The XP level in two orthogonal planes ($\varphi = 0^{ \circ }$ and $\varphi = 90^{ \circ }$) is compared and shown in Fig. 13c and d, respectivily. In the proposed metasurface antenna of Step #3, the XP level is considerably reduced, measuring below − 26 dB and − 48 dB in the $\varphi = 0^{ \circ }$ and $\varphi = 90^{ \circ }$ planes, respectively. These results clearly demonstrate that the performance of the antenna in Step #3 surpasses that of the first two steps.

**Figure 14:**
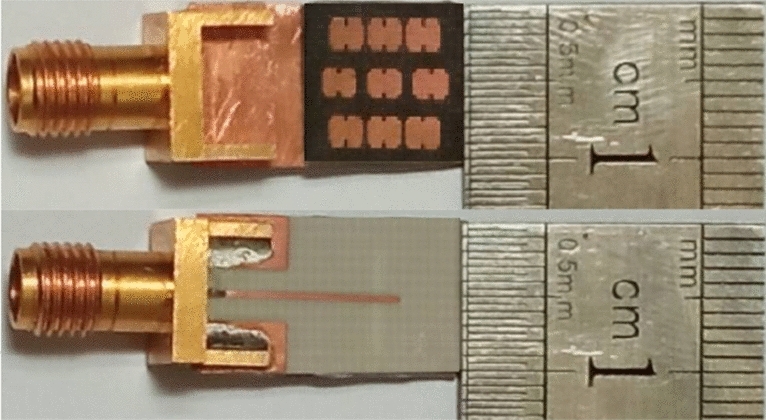
The fabricated proposed metasurface antenna.

**Figure 15:**
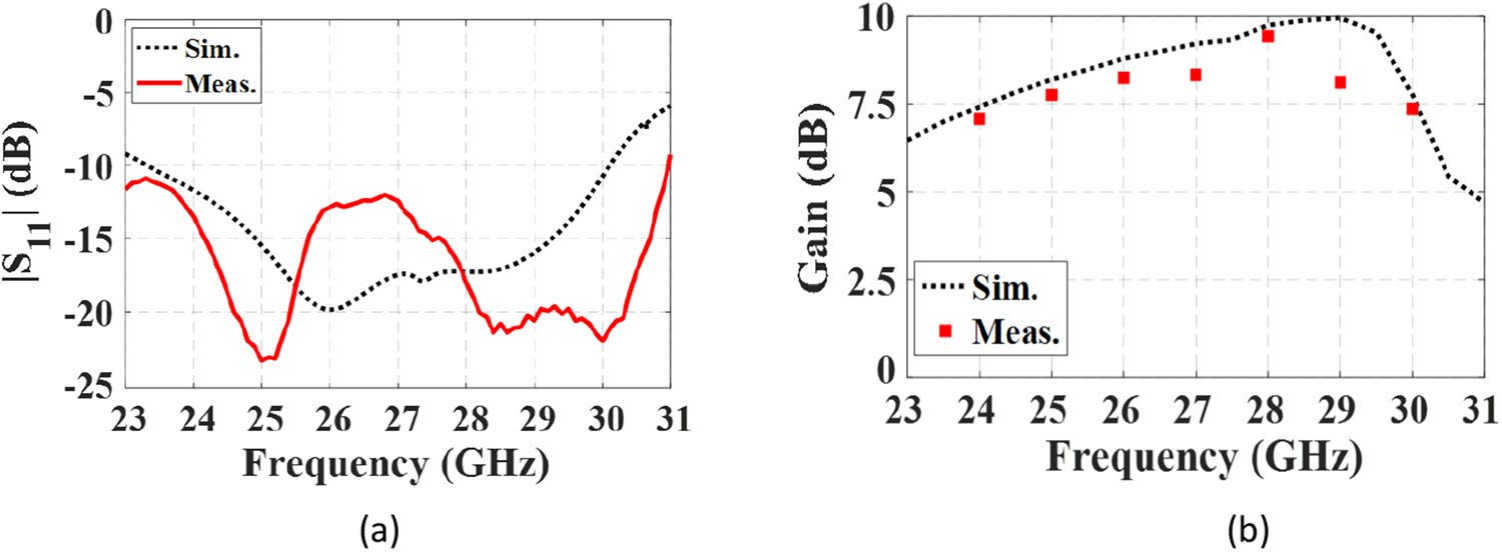
Simulated and measured results of the proposed metasurface antenna. (**a**) reflection coefficient, (**b**) variation of the gain at broadside direction vs. frequency.

**Figure 16:**
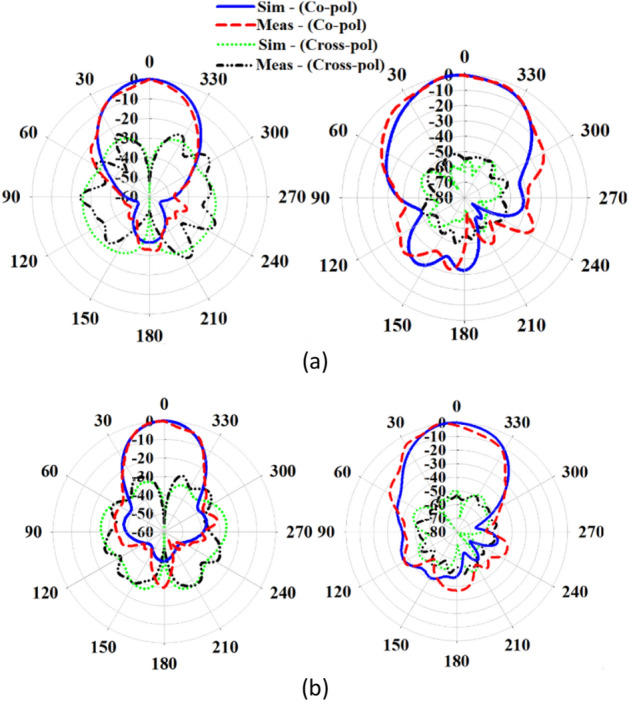
Simulated and measured normaized co- and cross-pol. radiation patterns of the proposed antenna in the $\varphi = 0^\circ$ (left) and $\varphi = 90^\circ$ (right) planes at (**a**) 26 GHz, (**b**) 28 GHz.

Figure 14 shows the fabricated prototype of the proposed final antenna. The measured reflection coefficient of the antenna is compared to the simulated results in Fig. 15a. The measured bandwidth is 29.6% (23–31 GHz) at |S_11_|< − 10 dB. A discrepancy between the simulated and measured results is observed, which may be due to fabrication tolerances, such as the effect of non-uniform gluing of the two layers. During assembly, the layers are glued along the corners, introducing air gaps that could create a potential difference between the simulation and measured results. Figure 15b illustrates variations in the broadside gain versus frequency, both simulated and measured. The measured maximum gain is 9.43 dB, with an efficiency exceeding 87%. Additionally, Fig. 16 displays the normalized co- and cross-pol. components of radiation patterns at the frequencies of 26 GHz and 28 GHz. The radiation patterns remain stable throughout the bandwidth, and the measured XP level is better than − 26 dB and − 48 dB in the $\varphi = 0^{ \circ }$ and $\varphi = 90^{ \circ }$ planes, respectively. The asymmetrical structure of the metasurface antenna contributes to the increase of the XP level in the $\varphi = 0^{ \circ }$ plane. It is worth noting that modifying the surface currents' directions of mode 5 to be orthogonal to mode 1, and relocating the maximum currents of modes 2, 3, and 4, significantly reduce the impact of higher-order modes on the antenna performance when mode 1 is excited. This results in a significant reduction in the XP level and an increase in the antenna's gain. The proposed design effectively addresses the challenges associated with higher-order modes, making it well-suited for applications in 5G mm-wave MIMO systems, where low XP level and enhanced performance are essential. The integration of the metasurface antenna with 5G mm-wave MIMO systems presents a promising solution for achieving high data rates and improved system capacity.

Table 2 presents a comparison of the proposed antenna with similar designs found in the literature. The proposed antenna demonstrates superior performance in terms of several key parameters, including wider bandwidth, higher efficiency, and significantly lower XP levels compared to other similar antennas, even with smaller dimensions. This simplified approach ensures independent mode performance, effectively preventing any interference between modes and resulting in outstanding antenna characteristics. The combination of optimized mode excitation, mode manipulation, and metasurface design allows the proposed antenna to achieve exceptional performance, making it a promising candidate for advanced 5G and beyond communication systems.

**Table 2:** A performance comparison between the proposed antenna and similar metasurface antennas.

Refs	Size (${\lambda }_{0}^{3}$)	Peak Gain (dB)	Bandwidth (%)	XP (dB)
^7^	1.07 × 1.07 × 0.06	9.8	28	< − 20
^8^	1.13 × 1.13 × 0.044	10	25.7	< − 20
^12^	1.03 × 1.03 × 0.023	10.1	16	< − 16
^13^	2.26 × 1.41 × 0.11	11.5	6.1	< − 10
^14^	1 × 1 × 0.072	10.3 (directivity)	31	Not reported
^21^	0.85 × 0.85 × 0.038	8.1	15.5	Not reported
This work	0.9 × 0.9 × 0.08	9.43	29.6	$\varphi = 90^\circ$ plane: < − 48 $\varphi = 0^\circ$ plane: < − 26

## Evaluation of the proposed antenna performance in MIMO configurations

**Figure 17:**
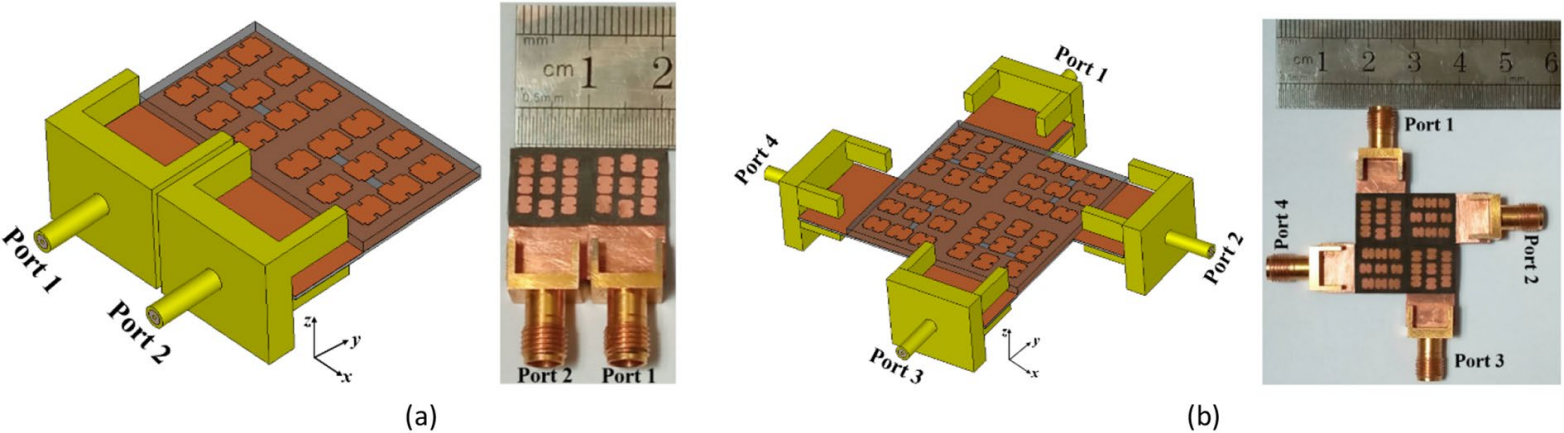
Simulated and fabricated MIMO antennas. (a) 1 × 2 configuration, (b) 2 × 2 configuration.

MIMO systems play a vital role in a wide range of applications in the 5G era. They are extensively used in various scenarios such as base station antennas, mobile phones, vehicle-to-vehicle (V2V) communications, and IoT devices^43^. The developed antenna is a promising candidate for MIMO applications due to its ability to generate independent beams for each metasurface element. This ensures high isolation between antennas when multiple elements are grouped together. To demonstrate its capabilities, two MIMO antennas with 1 × 2 and 2 × 2 configurations are designed. In the 1 × 2 MIMO configuration, the antenna elements are placed side-by-side to evaluate their ability to support identical polarizations. On the other hand, the 2 × 2 MIMO configuration involves placing the antennas with a 90° rotation relative to each other to assess the capability of the proposed antenna in supporting polarization diversity. In both configurations, the elements are positioned edge-to-edge to minimize dimensions and enhance compactness. The simulated layouts and the manufactured prototypes for 1 × 2 and 2 × 2 MIMO configurations are depicted in Fig. 17a and b, respectively. The practical evaluation of these prototypes will provide valuable insights into their real-world performance, including their ability to achieve high isolation and maintain stable radiation patterns. This validation process will further establish the suitability of the proposed metasurface antenna for practical MIMO applications in 5G and future communication systems.

**Figure 18:**
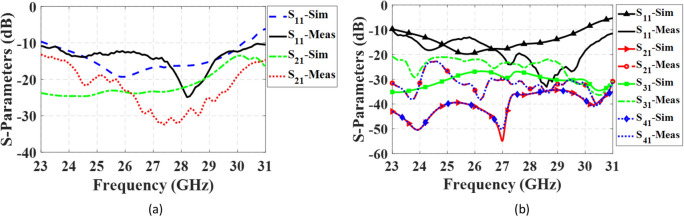
Simulated and measured S-parameters of MIMO configurations when port 1 is excited. (**a**) 1 × 2 configuration, (**b**) 2 × 2 configuration.

**Figure 19:**
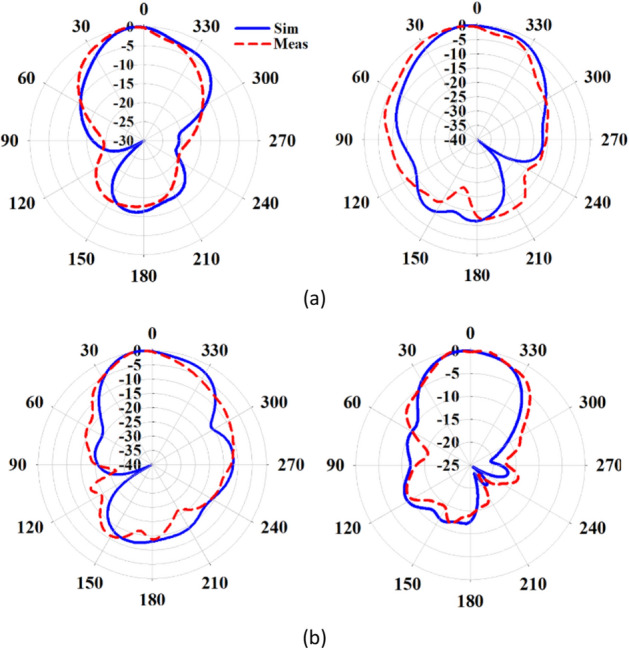
Normalized radiation patterns of the proposed metasurface antenna in the 1 × 2 MIMO configuration when port 1 is excited in the $\varphi = 0^\circ$ (left) and $\varphi = 90^\circ$ (right) planes at (**a**) 26 GHz, (**b**) 28 GHz.

**Figure 20:**
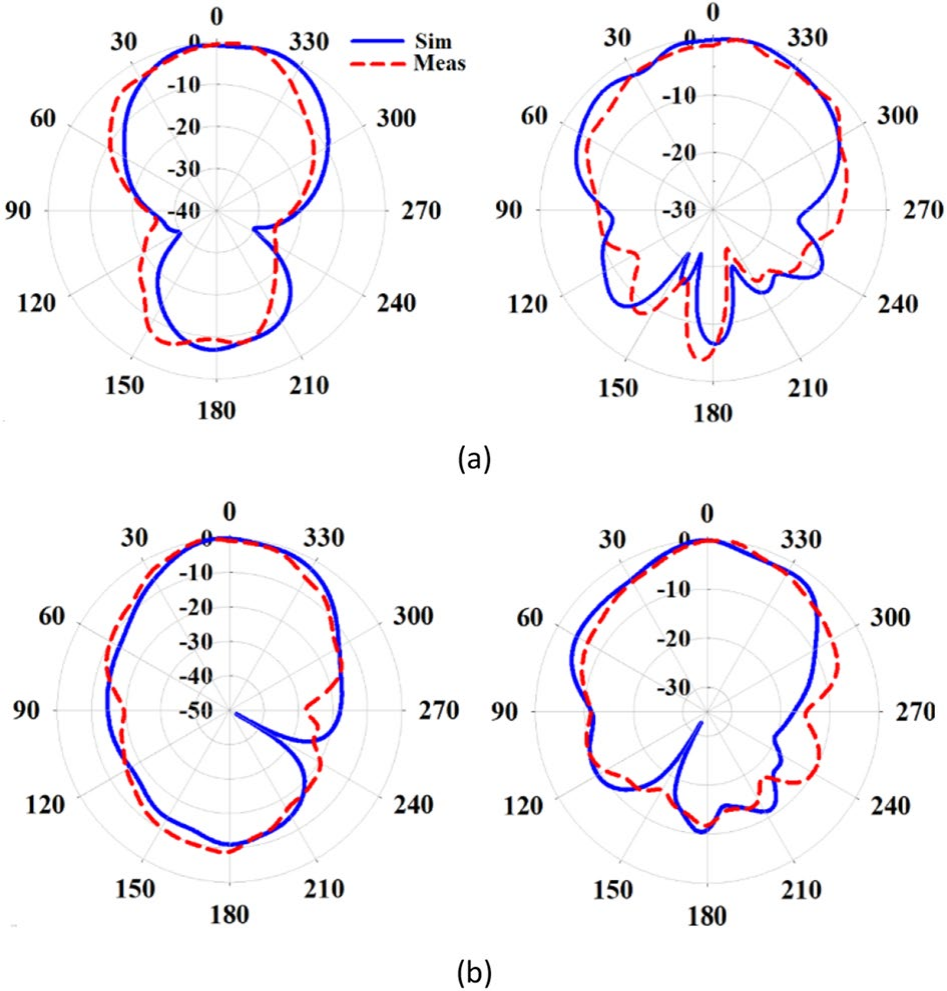
Normalized radiation patterns of the proposed metasurface antenna in the 2 × 2 MIMO configuration when port 1 is excited in the $\varphi = 0^\circ$ (left) and $\varphi = 90^\circ$ (right) planes at (**a**) 26 GHz, (**b**) 28 GHz.

The simulated and measured S-parameters for both configurations are presented in Fig. 18. In the 1 × 2 and 2 × 2 MIMO configurations, the isolation between adjacent ports exceeds 18 dB and 21 dB, respectively, across the entire operating frequency range from 24.5 to 29.5 GHz. This high level of isolation indicates that the proposed antenna maintains excellent independence between the elements, ensuring minimal interference and crosstalk in the multi-port system. Figures 19 and 20 depict the simulated and measured radiation patterns of the MIMO antennas in the 1 × 2 and 2 × 2 configurations, respectively, at frequencies of 26 GHz and 28 GHz. The proposed antenna exhibits stable and smooth radiation patterns in both configurations, remaining almost broadside throughout the entire bandwidth. This confirms that the antenna performance in the MIMO configurations is comparable to that of the isolated single-element antenna, effectively preserving the radiation characteristics even when adjacent elements are present.

**Figure 21:**
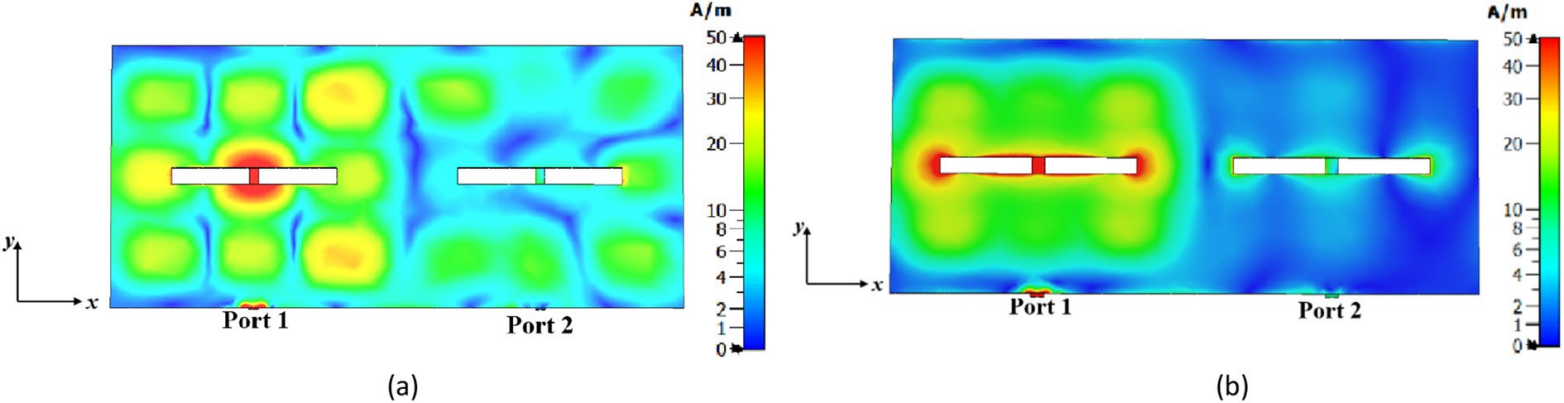
The surface current distribution in the 1 × 2 MIMO configuration at 27 GHz when port 1 is excited and port 2 is matched. (**a**) conventional metasurface antenna (Step #1), (**b**) proposed metasurface antenna (Step #3).

Figure 21a and b illustrate the surface currents of the 1 × 2 MIMO antennas at 27 GHz for two cases: conventional (Step #1) and proposed (Step #3) metasurface antennas, respectively. In both cases, port 1 is excited, and port 2 is 50 Ω matched. As can be seen, the conventional metasurface antenna exhibits strong coupling from port 1 to port 2 due to the presence of higher-order modes. In contrast, the proposed metasurface antenna effectively addresses this challenge with suppressing the effects of higher-order modes.

**Figure 22:**
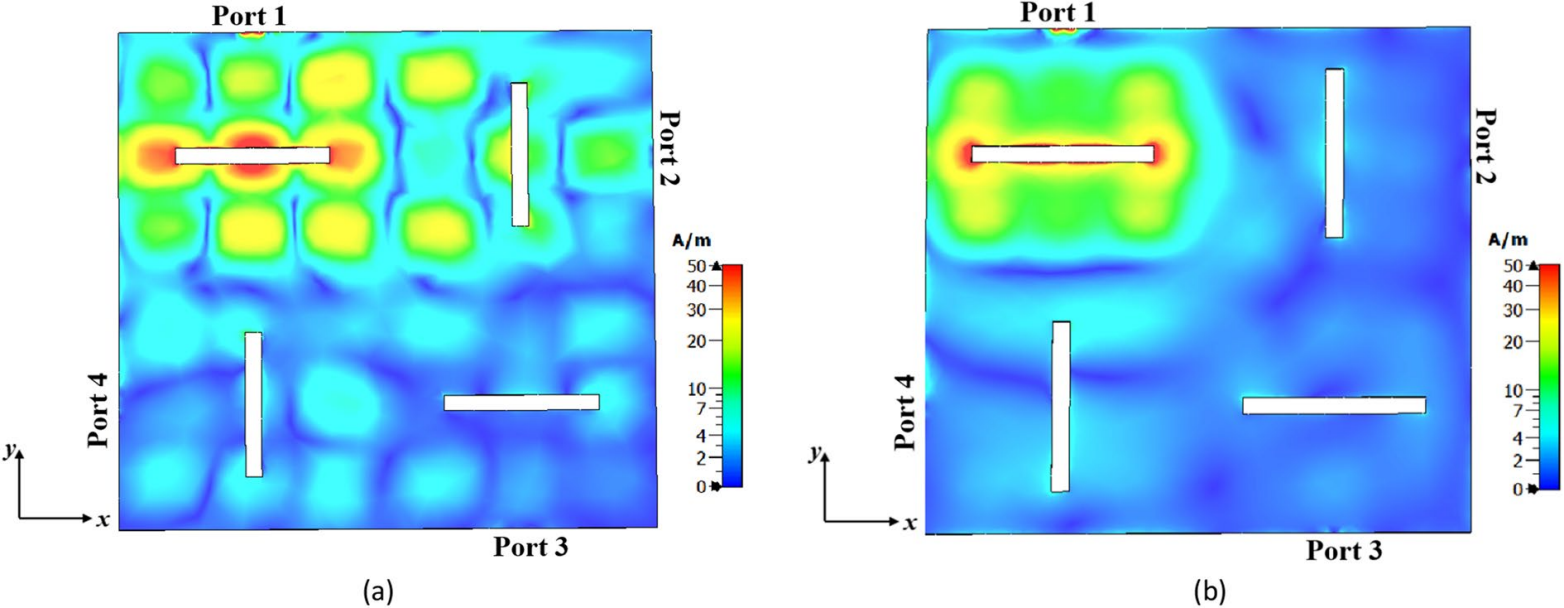
The surface current distribution in the 2 × 2 MIMO configuration at 27 GHz when port 1 is excited and other ports are matched. (**a**) Conventional metasurface antenna (Step #1), (**b**) proposed metasurface antenna (Step #3).

In the 2 × 2 MIMO configuration, the antennas are arranged with a 90° rotation relative to each other, contributing to the decrease in mutual coupling between ports. Figure 22a and b illustrate the surface currents of conventional and proposed metasurface antennas in the 2 × 2 MIMO configuration, respectively, with port 1 being excited while the other ports are 50 Ω matched. It is evident that the coupled currents from port 1 to the other ports in the proposed metasurface antenna exhibit a significant reduction. This reduction can be attributed to the effectiveness of the proposed metasurface antenna in suppressing higher-order modes. As a result, the elimination of unwanted higher-order modes in the desired frequency range also plays a significant role in reducing coupling between antenna ports.

In the evaluation of the radiation patterns' independence for the MIMO antennas, two metrics are employed: ECC and DG. ECC is utilized to measure the correlation between the 3D radiation patterns of the antennas and is typically desired to be less than 0.5^44^. The ECC between antennas #*m* and #*n* can be calculated using the following equation (Eq. 6)^2^:

6 $${\text{ECC}}_{mn} = \frac{{\left| {\int {\int\limits_{0}^{4\pi } {[\vec{F}_{m} (\theta ,\phi ) \times \vec{F}_{n} (\theta ,\phi )]d\Omega } } } \right|^{2} }}{{\int {\int\limits_{0}^{4\pi } {\left| {\vec{F}_{m} (\theta ,\phi )} \right|^{2} d\Omega \int {\int\limits_{0}^{4\pi } {\left| {\vec{F}_{n} (\theta ,\phi )} \right|^{2} d\Omega } } } } }}$$

where, $\vec{F}_{m} (\theta ,\phi )$ and $\vec{F}_{n} (\theta ,\phi )$ represent the 3D radiation patterns of antennas *#m* and *#n*, respectively, and $d\Omega$ is the differential solid angle. The second parameter, DG, is calculated using Eq. (7)^45^:

7 $${\text{DG(dB)}} = 10\sqrt {1 - \left| {{\text{ECC}}_{mn} } \right|^{2} }$$

**Figure 23:**
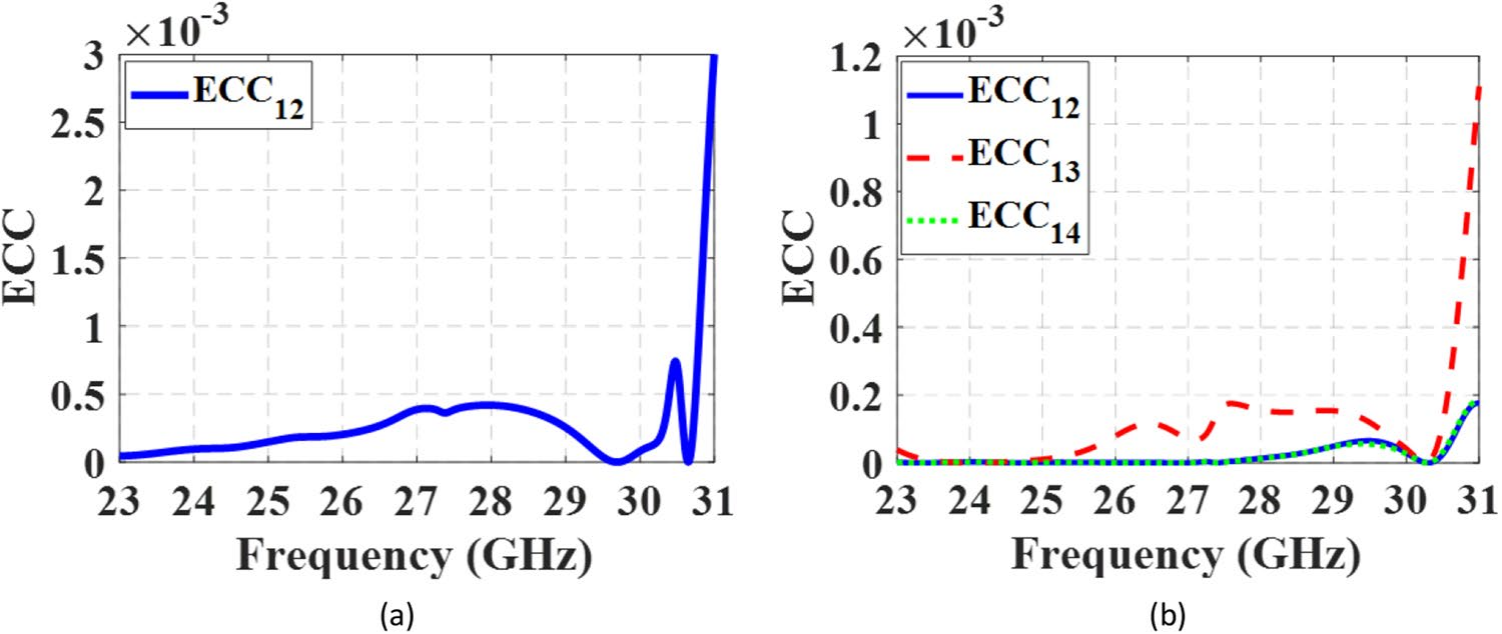
The ECCs of the MIMO antennas. (**a**) 1 × 2 configuration, (**b**) 2 × 2 configuration.

Figure 23a and b depict the ECCs of the 1 × 2 and 2 × 2 MIMO antennas, respectively. The ECC values are below 0.003 for the 1 × 2 configuration and below 0.0011 for the 2 × 2 configuration across the entire frequency range, indicating exceptional diversity performance of the proposed antenna. Additionally, the MIMO antennas exhibit high DGs, with values exceeding 9.997 dB and 9.994 dB for the 1 × 2 and 2 × 2 configurations, respectively, throughout the operating frequency band.

In Table 3, a comparison of the performance of the proposed antenna in the 1 × 2 MIMO configuration with other antennas is presented. It is worth nothing that the MIMO antennas mentioned in^30–34^ are smaller in size compared to our proposed antenna. However, the antennas in^31–33^ suffer from a significant beam squint angle, which is a notable drawback in MIMO setups. Furthermore, their radiation patterns are unstable, making them less reliable. The previous studies primarily focused on improving isolation between ports, but the methods employed were not effective in maintaining the desired direction of radiation patterns. As a result, these antennas are not well-suited for 5G mm-wave massive MIMO applications. On the other hand, the antennas in^30,34^ have stable radiation patterns, but they encounter challenges related to a narrow bandwidth. In the design of our metasurface-based antenna for the 5G mm-wave band, we have taken great care to strike a balance between size and addressing the main issues, such as the beam squint angle, stable radiation patterns, and wider bandwidth.

**Table 3:** Performance comparison between proposed antenna in a 1 × 2 MIMO configuration and other works.

Refs	Center-to-center spacing (λ_0_)	1 × 2 MIMO size (${\lambda }_{0}^{3}$)	Center frequency (GHz)	Bandwidth (%)	Isolation (dB)	Stable radiation patterns	Beam squint (°)	Complexity	Design method
^30^	0.51	1.1 × 0.51 × 0.07	29	3.7	> 36	Yes	0	Low	Bending rectangular patch antennas with two slits in each antenna
^31^	0.1	0.5 × 0.5 × 0.18	10	27.8	> 18	No	42	High; using air-gap and one dielectric block with high permittivity	A dielectric block above the array
^32^*	0.35	1.16 × 0.75 × 0.18	3.5	2.85	> 20	No	30	Medium; using air-gap	Array of slot metasurface above the patch antennas
^33^	0.58	1.44 × 0.9 × 0.05	5.4	14.8	> 20	No	30	Medium; using parasitic elements	Multiple square parasitic elements in proximity of the rectangular patch antennas
^34^	0.375	1.58 × 0.81 × 0.13	4.9	6	> 20	Yes	0	Low; using strips between elements	Field superposition
This work	0.9	1.8 × 0.9 × 0.08	27	29.6	> 18	Yes	0	Low	Manipulating the surface currents of the modes

*Simulated results.

λ_0_: is the free space wavelength at the center frequency.

Furthermore, Table 4 presents a comparison between the performance of the proposed metasurface antenna in the 2 × 2 MIMO configuration and other structures reported in previous studies. The results show that the proposed metasurface antenna exhibits significantly reduced beam squint angles in its radiation patterns. Additionally, the proposed structure boasts low complexity, making it a practical and efficient solution for MIMO applications.

**Table 4:** Performance comparison of proposed metasurface antenna with other antennas in a 2 × 2 MIMO configuration.

Refs	Center-to-center spacing (λ_0_)	2 × 2 MIMO size (${\lambda }_{0}^{3}$)	Center frequency (GHz)	Bandwidth (%)	Isolation (dB)	Beam split	Beam squint (°)	Complexity	Design method
^18^	0.73	1.46 × 1.46 × 0.09	5.5	21	> 26	No	(2)–(8)	High; using metal pins	Suppressing higher-order modes
^35^	0.5	1.06 × 1.06 × 0.03	5.8	3.4	> 32	No	xy-plane: (− 8)–(− 4) yz-plane: (17)–(28)	Low; modifying the ground plane	Defected ground structure (DGS)
^36^	1.12	2.08 × 2.08 × 0.37	26	21.1	> 22	Yes	(− 30)–(30)*	Medium; using air-gap	Metasurface with circular shaped unit cell placed in back side of the antennas
^37^	1.47	3.72 × 2.60 × 0.63	26	7.5	> 35	Yes	(− 16)–(25)	Medium; using air-gap	A metasurface with Circular Split Ring (CSR)
^46^	0.71	1.87 × 1.87 × 0.04	27.5	16.3	> 30	No	Not reported	Low	A metasurface with a 2 × 2 periodic square metallic plates
^47^	0.735	1.47 × 1.47 × 0.07	27.63	21.7	> 27	No	0	Low	Binomial series-fed array with loaded patches
^48^	0.88	2.24 × 2.24 × 0.68	28	20	> 30	Yes	E-plane: (0)–(15) H-plane: (0)–(25)	Medium; using air-gap	Partially Reflective Surface (PRS)
This work	0.9	1.8 × 1.8 × 0.08	27	29.6	> 21	No	0	Low	Manipulating the surface currents of the modes

*Estimated from the reported figures.

λ_0_: is the free space wavelength at the center frequency.

## Conclusion

This paper presents an innovative methodology for designing compact and low cross-polarization (XP) metasurface antennas specifically tailored for 5G mm-wave MIMO systems. Unlike traditional symmetric metasurfaces, this novel design eliminates the need for inter-element spacing. This achievement is made possible through the utilization of the CMA technique, which allows for the manipulation of various modes and the engineering of their characteristics. The proposed design comprises a 3 × 3 array of asymmetric patches, each featuring two narrow rectangular slits on their sides and two stubs on the top and bottom. These modifications serve multiple purposes, including altering the direction and redistributing surface currents of higher-order modes, separating degenerate modes, and reshaping the radiation patterns of the fundamental mode at higher frequencies towards broadside. Furthermore, compared to conventional patch metasurface antennas, this novel design achieves a significant size reduction of approximately 41%. Measurements of the proposed metasurface antenna indicate a maximum gain of 9.43 dB at 28 GHz. Moreover, the XP level is below − 26 dB and − 48 dB across the entire operating frequency band (23–31 GHz) in $\varphi = 0^{ \circ }$ and $\varphi = 90^{ \circ }$ planes, respectively.

To validate the effectiveness of the proposed design in suppressing undesirable higher-order modes, two types of MIMO antennas were manufactured and thoroughly tested. The first setup involved a 1 × 2 configuration with adjacent antenna elements placement, which was used to evaluate the performance of identical polarization. The second setup utilized a 2 × 2 arrangement with a 90° rotational configuration to evaluate polarization diversity. Both setups demonstrated stable radiation patterns and effectively addressed common issues encountered in conventional metasurface patch antennas, such as radiation pattern ripples, beam splitting, and beam squint. In conclusion, the proposed metasurface antenna offers a compelling and practical solution for compact 5G mm-wave massive MIMO applications, promising improved performance and enhanced capabilities for future communication systems.

## Data Availability

The datasets generated during and/or analyzed during the current study are available from the corresponding author on reasonable request.
